# The crucial role of N6-methyladenosine modification in acute kidney injury: mechanisms and therapeutic potential

**DOI:** 10.3389/fimmu.2026.1864973

**Published:** 2026-07-13

**Authors:** Xuechao Zhang, Xiaoshan Chen, Lizhu Yang, Yanan Jin, Weiran Zhang, Jinge Zhang, Jiaguo Huang

**Affiliations:** 1 Department of Urology, Baoshan People’s Hospital, Baoshan, Yunnan, China; 2 Ultrasound Department, Baoshan People’s Hospital, Baoshan, Yunnan, China; 3 Department of Urology, Affiliated Xiaoshan Hospital, Hangzhou Normal University, Hangzhou, China

**Keywords:** acute kidney Injury, inflamation, N6-methyladenosine, programmed cell death, therapeutic targets

## Abstract

Acute kidney injury (AKI) is a clinically critical condition with a high mortality rate. Its complex pathophysiological mechanisms remain incompletely understood, and there is a lack of effective targeted therapeutic strategies. In recent years, the role of epigenetic modifications in the initiation and progression of kidney disease has been increasingly clarified. N6-methyladenosine (m6A) is one of the most important and common post-transcriptional modifications among various RNAs, including eukaryotic mRNAs, lncRNAs, and miRNAs. It is dynamically and reversibly regulated by methyltransferases (‘writers’), demethylases (‘erasers’), and binding proteins (‘readers’) to modulate processes such as RNA splicing, export, stability, translation, and degradation. This modification exerts diverse biological effects and is extensively involved in both physiological and pathological pathways. Recently, a growing body of evidence has indicated that m6A modification plays a crucial regulatory role in the development and progression of AKI. Existing studies suggest that m6A modification profoundly influences the fate of renal tubular epithelial cells (TECs) by regulating the expression of genes associated with inflammatory responses and programmed cell death, thereby modulating the severity of AKI and the subsequent renal repair process. This review systematically summarizes the latest research advances regarding m6A modification in AKI, elucidates the mechanisms by which it influences the pathogenesis of AKI through various cellular processes, and explores the potential of m6A-targeted therapies for treating AKI, thereby providing insights into the regulatory networks of m6A modification in AKI and the epigenetic regulation of transcription in this condition.

## 1 Introduction

Acute kidney injury (AKI) is a common clinical syndrome characterized by a rapid decline in renal function and is closely associated with high morbidity, mortality, and the risk of progression to chronic kidney disease (CKD) (1). Its etiology is diverse, including ischemia/reperfusion, sepsis, and nephrotoxic drugs (e.g., cisplatin). Despite advances in supportive care, the pathophysiological mechanisms underlying AKI remain incompletely understood, which directly hampers the development of effective prevention and treatment strategies. In recent years, the emergence of epitranscriptomics—the study of post-transcriptional modifications of RNA transcripts—has opened entirely new perspectives for understanding the mechanisms of this complex disease.

Among the numerous post-transcriptional modifications of RNA, N6-methyladenosine (m6A), the most prevalent and abundant internal chemical modification in eukaryotic mRNA, plays a pivotal role in post-transcriptional regulation. The m6A modification is dynamically and reversibly regulated, precisely influencing the entire metabolic process of RNA, including splicing, nuclear export, stability, translation, and degradation, thereby playing a widespread role in biological processes such as cell fate determination, stress responses, and immune regulation (2). A growing body of evidence suggests that dysregulation of m6A modification is a major driver of the initiation and progression of cancer, neurodegenerative diseases, metabolic disorders, and cardiovascular diseases (3–5).

Notably, the kidney, as a highly metabolically active and vulnerable organ, relies on the precise and rapid reprogramming of gene expression in its cells (particularly renal tubular epithelial cells) when responding to injury; this process is central to determining whether cells survive, repair themselves, or undergo cell death. This observation suggests that m6A modification, as a key component of gene expression regulation, likely plays a crucial role in the pathological progression of AKI. Recent studies have preliminarily revealed that m6A modification profoundly influences the fate of renal tubular epithelial cells (TECs) and the outcome of AKI by regulating key signaling pathways such as inflammatory responses and programmed cell death.

This review aims to systematically summarize current research progress regarding the role and mechanisms of m6A modification in AKI. We explore in depth the role and mechanisms of m6A modification in AKI induced by different etiologies and discuss the potential and challenges of targeting m6A regulatory elements as novel diagnostic biomarkers and therapeutic strategies for AKI, with a view to providing a theoretical basis and new insights for future research directions.

## 2 Overview and pathophysiology of AKI

AKI is a clinical syndrome defined by a sharp decline in renal function within hours to days, primarily manifested as elevated serum creatinine and blood urea nitrogen levels and/or reduced urine output. AKI represents a global public health burden, affecting more than 55 million individuals annually. Worldwide, approximately 10–15% of hospitalized patients and up to 50% of intensive care unit (ICU) patients develop AKI (6) In the short term, AKI increases patient mortality, prolongs hospital stays, and consumes substantial healthcare resources. In the long term, it is closely associated with adverse outcomes, including chronic kidney disease, end-stage renal disease, cardiovascular disease, and an elevated risk of all-cause mortality (7) The etiology of AKI is complex and heterogeneous. Traditionally, it is classified into prerenal, intrinsic renal, and postrenal categories based on the anatomical site of insult. Its pathophysiology is multifaceted and dynamic, involving interactions among hemodynamic alterations, cellular injury, and inflammatory responses. Although the predominant mechanisms vary by etiology, common convergent pathways exist. Renal hypoperfusion serves as the initiating event in AKI. Under stress conditions, activation of the sympathetic nervous system and the renin-angiotensin-aldosterone system induces afferent arteriolar vasoconstriction and reduces the glomerular filtration rate. Concurrently, endothelial dysfunction diminishes the production of vasodilatory factors while increasing vasoconstrictors, further exacerbating intrarenal vasoconstriction and establishing a vicious cycle (8). TECs injury is a core event in AKI. Ischemia, hypoxia, or direct nephrotoxic insult disrupts energy metabolism, dismantles the cytoskeleton, and causes loss of cellular polarity in tubular epithelial cells, ultimately triggering programmed cell death and necrosis (9). Inflammation and immune responses constitute critical mechanisms driving AKI onset and progression. In the context of sepsis, pathogen-associated molecular patterns (PAMPs) and damage-associated molecular patterns (DAMPs) are released into the intravascular space, initiating downstream signaling cascades and activating the innate immune system. This leads to inflammatory cell infiltration and the production of abundant pro-inflammatory cytokines and chemokines, generating a “cytokine storm” that results in microvascular congestion, leukocyte adhesion, and further tissue damage (10, 11). In summary, the pathophysiology of AKI is highly complex. Renal hypoperfusion, excessive inflammatory responses, cellular necrosis, and programmed cell death play central roles in its pathogenesis. These mechanisms are mutually reinforcing and collectively drive the initiation and progression of AKI.

Notably, recent studies have increasingly emphasized the role of programmed cell death of TECs in AKI. Multiple forms of cell death have been reported in AKI, including apoptosis, pyroptosis, ferroptosis, and autophagy, each characterized by distinct molecular features and regulatory mechanisms. Accumulating experimental evidence supports the pathogenic role of apoptosis in AKI (12, 13). Proximal tubular epithelial cells are particularly susceptible to apoptosis, and injury in this segment can precipitate AKI and organ failure (14). Pyroptosis is a pro-inflammatory form of programmed cell death characterized by the formation of plasma membrane pores, cellular swelling, membrane lysis, release of inflammatory mediators, and activation of innate immunity, ultimately leading to cell death (15). Numerous studies have highlighted the critical role of pyroptosis in extensive tissue injury, driven by robust inflammasome activation and secretion of pro-inflammatory cytokines. Once activated, inflammasomes recruit and activate caspase-1 or caspase-11, which subsequently cleave Gasdermin D (GSDMD) (16). Cleavage of GSDMD is a pivotal event in pyroptosis, as the N-terminal fragment oligomerizes to form pores in the cell membrane, triggering cell lysis and the release of intracellular contents (17, 18). The kidney is a major target organ of pyroptosis-mediated injury, and extensive research underscores its key role in the pathogenesis of AKI (19–21). Iron is an essential trace element with multiple critical biological functions. However, excessive intracellular iron, particularly ferrous overload, can induce lipid peroxidation. In 2012, Dixon et al. first formally defined this iron-dependent, lipid peroxidation-driven form of regulated cell death as ferroptosis (22). Key hallmarks of ferroptosis include lipid peroxidation and iron accumulation, which lead to inactivation of glutathione peroxidase 4 (GPX4) and subsequent cell death (23). Ferroptosis is closely implicated in the pathogenesis and progression of various diseases, including ischemia-reperfusion injury, kidney injury, neurological disorders, cancer, and hematological diseases. Expression of ferroptosis-related signals correlates positively with AKI incidence and mortality. Conversely, ferroptosis inhibitors exert renoprotective effects in diverse AKI animal models, underscoring the pivotal role of ferroptosis in AKI pathogenesis (24, 25). Autophagy is a conserved lysosomal degradation pathway for cytoplasmic components, essential for maintaining renal homeostasis, structure, and function. Conversely, dysregulated autophagy contributes to AKI and represents an important mechanism underlying incomplete renal repair following injury (26). For example, following AKI, TECs produce fibroblast growth factor 2 (FGF2) via autophagy, leading to fibroblast activation and subsequent renal fibrosis (27).

Due to the complexity of the pathophysiological mechanisms of AKI, the intricacy of the clinical research process, and certain technical and ethical constraints, progress in understanding the pathogenesis of AKI remains slow and limited, and the treatment of AKI remains challenging (28). The selection of stable and appropriate preclinical models can help more accurately simulate the clinical features of AKI. Currently, the main AKI models include ischemia-reperfusion(I/R) injury-induced AKI, cisplatin-induced AKI, sepsis-induced AKI, folic acid-induced AKI, and rhabdomyolysis-induced AKI (29, 30). Among these, the two most commonly used methods for modeling experimental septic AKI are cecal ligation and puncture (CLP) surgery and lipopolysaccharide (LPS) injection (31–33).

## 3 m6A modification

**Figure 1:**
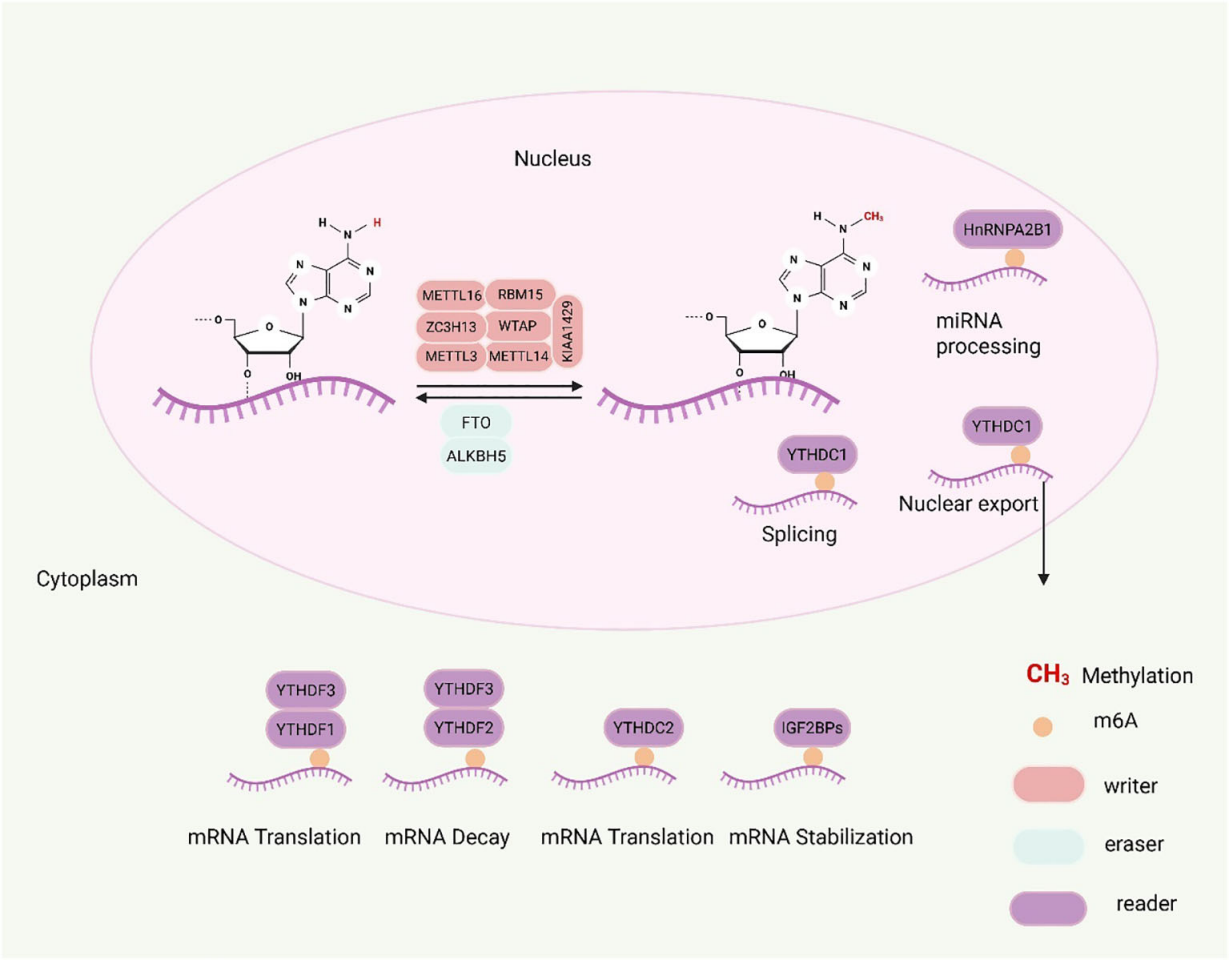
Mechanisms of m6A modification. RNA undergoes m6A modification via addition of a methyl group to the N6 position of adenine through the action of “writers.” This modification is reversible and can be reversed by “erasers.” In the presence of “readers,” m6A-modified RNA is directed toward various fates, including splicing, nuclear export, translation, and enhanced stability, which ultimately influence the expression of target genes.

As an important branch of epigenetics, epitranscriptomics has revealed that over 150 types of RNA chemical modifications have been identified to date (34, 35). Among the more than 100 RNA modifications discovered, m6A is the most common and abundant internal chemical modification in eukaryotic mRNAs; approximately 25% of all cellular transcripts contain multiple m6A-modified residues. This modification involves the covalent addition of a methyl group to the N6 position of adenine. This process dynamically and reversibly regulates gene expression at the post-transcriptional level and is regarded as an important frontier in epigenetic regulation, complementing DNA methylation and histone modifications. m6A modification occurs primarily within RRACH sequences (where R = A or G, and H = A, C, or U) and is mainly enriched near stop codons, specifically in the 3′ untranslated region (3′UTR) as well as in long introns and exons (36–39). Furthermore, it is also present in pre-mRNA and non-coding RNAs (40, 41). m6A modification is dynamic and reversible; its establishment, recognition, and removal are precisely executed by a highly conserved protein complex, often metaphorically described as “writers,” “erasers,” and “readers.” “Writers” catalyze the formation of m6A modifications (**Figure 1**). Their core consists of a heterodimeric complex formed by methyltransferase-like 3 (METTL3) and methyltransferase-like 14 (METTL14), in which METTL3 is the primary catalytic subunit, whereas METTL14 is primarily responsible for recognizing RNA substrates and maintaining the stability of the complex. Furthermore, co-factors such as Wilms tumor-associated protein 1 (WTAP) are crucial for guiding the complex to localize at nucleolar sites and for maximizing its catalytic activity. Additionally, the “writers” of m6A include METTL16, KIAA1429, RBM15, VIRMA, and ZC3H13 (40, 42, 43). “Erasers” are responsible for removing m6A modifications, thereby ensuring their reversibility. The two best-characterized demethylases are fat mass and obesity-associated protein (FTO) and AlkB-homologous protein 5 (ALKBH5). Through oxidative demethylation reactions, they dynamically “erase” m6A marks, thereby finely regulating the fate of mRNA. “Readers” recognize and bind to m6A modifications, thereby mediating downstream biological functions. This class of proteins is functionally diverse and exhibits a wide range of functions. The most well-known m6A “readers” are the YTHDF family, the YTHDC family, and the IGF2BP family (44). The YTHDF family (e.g., YTHDF1/2/3) is primarily located in the cytoplasm and regulates gene expression by promoting mRNA translation (YTHDF1), accelerating mRNA degradation (YTHDF2), or influencing both translation and degradation (YTHDF3) (36, 45). The YTHDC family (e.g., YTHDC1/2), including the nuclear protein YTHDC1, is primarily involved in mRNA splicing and export from the nucleus; YTHDC1 enhances translation efficiency but reduces the abundance of the target mRNA (46, 47). The IGF2BP family recognizes m6A and, through distinct mechanisms, enhances the stability and translation efficiency of target mRNAs (48).

Through the synergistic action of the aforementioned regulatory elements, m6A modification exerts a wide-ranging influence on the entire life cycle of mRNA, including pre-mRNA splicing, nuclear-cytoplasmic transport, mRNA stability, translation efficiency, and degradation. Consequently, m6A modification plays a crucial role in cell differentiation, embryonic development, circadian rhythms, the DNA damage response, and the initiation and progression of various diseases. In recent years, its role in kidney diseases, particularly AKI, has emerged as a significant research focus, revealing novel functions of this post-transcriptional regulatory mechanism in renal stress and repair.

## 4 m6A Modification in AKI

As an important epigenetic modification, m6A plays a key role in the initiation and progression of kidney disease. In various AKI models, alterations in m6A modification levels and the expression of its regulatory proteins have been observed, suggesting that m6A dysregulation may represent a critical mechanism driving AKI onset and progression (49) (**Table 1**). METTL3 is involved in maintaining the homeostasis of TECs under physiological conditions. However, in AKI, METTL3 expression and global m6A levels are persistently elevated in renal tissue, particularly within TECs Studies have reported increased METTL3 expression in multiple AKI mouse models, including those induced by cisplatin, LPS, and I/R, leading to renal inflammation and cell death (50). Another methyltransferase component, ZC3H13, is also significantly upregulated in cisplatin-induced AKI, where it induces G2/M cell cycle arrest and apoptosis (51). METTL14, another key methyltransferase, has been reported to be upregulated in LPS-treated TCMK-1 cells and cisplatin-treated HK-2 cells, promoting ferroptosis and AKI progression (52, 53). However, conflicting evidence exists: METTL14 has also been found to be significantly downregulated in renal tissues from human sepsis-associated AKI patients and in CLP- and I/R-induced AKI mouse models, and restoring its expression improves renal function (54, 55).

**Table 1:** Expression patterns and functional roles of m6A regulators in various models of AKI.

AKI model	Cell type	m6A regulator	Expression	Target	Key findings	Reference
Cisplatin, LPS, and I/R-induced AKI	HK-2, mTECs	METTL3, IGF2BP2	Upregulated	TAB3	METTL3 promotes renal inflammation and programmed cell death	(50)
Lead acetate-induced AKI	HK2	METTL3	Upregulated	HKDC1	METTL3 promotes renal injury and inflammation	(63)
Ischemic, septic, and vancomycin-induced AKI	BUMPT	METTL3	Upregulated	mmu-lncRNA 121686/hsa-lncRNA 520657	METTL3 promotes AKI via the lncRNA 121686/miR-328-5p/HtrA3 axis	(64)
CLP- induced AK	RAW264.7	FTO	Downregulated	MMP-9	FTO reduces M1 macrophage polarization and attenuates LPS-induced inflammation	(65)
CLP- induced AKI	HK2	IGF2BP1	Upregulated	E2F1	IGF2BP1 targets the MIF component of NLRP3 inflammasome, acting as a potent pyroptosis inducer in septic AKI	(61)
CLP- induced AKI	HK-2	METTL3, YTHDF1	Upregulated	MDM2	METTL3-mediated m6A modification regulates the MDM2-p53-LMNB1 axis, stimulating mitochondrial damage and ferroptosis in TECs	(66)
CLP- induced AKI	HK-2	METTL14	Downregulated	HMOX1	METTL14 inhibits ferroptosis and protects against AKI via m6A-dependent regulation of HMOX1	(54)
CLP- induced AKI	HK2	HNRNPC	Upregulated	NF-κB p65	HNRNPC-mediated m6A drives tubular dysfunction in sepsis-induced AKI	(67)
CLP- induced AKI	Mouse microvascular pericytes	MTHFD2	Upregulated	LOX	MTHFD2 promotes LOX expression in an m6A-dependent manner, mediating AKI progression	(68)
LPS- induced AKI	TCMK-1	METTL3	Upregulated	MALAT1	METTL3 promotes inflammatory cytokine release and pyroptosis in AKI by enhancing m6A modification of MALAT1	(69)
LPS- induced AKI	TCMK-1	RBM15 and IGF2BP1	Upregulated	CRBN	RBM15 increases CRBN m6A modification and promotes its expression in an IGF2BP1-dependent manner, enhancing pyroptosis in TECs	(70)
LPS- induced AKI	HK-2	WTAP	Upregulated	LMNB1	WTAP-mediated m6A promotes LPS-induced inflammation, mitochondrial damage, and ferroptosis by regulating LMNB1 expression and activating NF-κB and JAK2/STAT3 pathways	(71)
LPS- induced AKI	TCMK-1	METTL14	Upregulated	LPCAT3	METTL14 promotes ferroptosis in septic AKI by increasing m6A methylation of LPCAT3	(52)
LPS- induced AKI	HK2	FTO	Downregulated	SNHG14	FTO regulates LPS-induced cell viability, apoptosis, and autophagy via the SNHG14/miR-373-3p/ATG7 interaction network	(56)
LPS- induced AKI	HK-2	ALKBH5	Upregulated	pri-miR-205	ALKBH5 suppresses miR-205-5p expression by removing m6A modification, thereby upregulating DDX5 and promoting EMT and the AKI-to-CKD transition	(72)
I/R- induced AKI	mRTEC	ALKBH5	Upregulated	CCL28	ALKBH5 inhibits renal Treg recruitment, increases macrophage and neutrophil infiltration, and promotes inflammation	(58)
I/R- induced AKI	Mouse TECs	METTL14, IGF2BP2	Downregulated	PPARγ	METTL14 regulates PPARγ m6A methylation in an IGF2BP2-dependent manner, playing a crucial role in alleviating renal I/R injury and TEC ferroptosis	(55)
I/R- induced AKI	HK-2, TCMK-1	METTL3	Upregulated	miR-374b-5p	METTL3 promotes renal I/R injury by regulating the miR-374b-5p/SRSF7 axis	(73)
I/R- induced AKI	HK2	METTL3 and IGF2BP2	Upregulated	TIFA	METTL3 enhances TIFA mRNA stability in an IGF2BP2-dependent manner, thereby promoting pyroptosis	(74)
I/R- induced AKI	HK-2	FTO	Downregulated	Ambra1	FTO promotes autophagy by upregulating the Ambra1/ULK1 signaling pathway, thereby inhibiting oxidative stress and renal injury	(57)
Cisplatin and I/R-induced AKI	HK2	YTHDF1	Upregulated	SHPK1	YTHDF1 alleviates AKI by protecting m6A-methylated mRNAs within renal tubular stress granules	(60)
Cisplatin - induced AKI	HK-2	METTL14、IGF2BP3	Upregulated	ERFE	METTL14 stabilizes ERFE mRNA through IGF2BP3-dependent m6A modification, promoting ferroptosis in cisplatin-induced AKI	(53)
Cisplatin - induced AKI	HK-2	ALKBH5	Upregulated	—	ALKBH5 promotes cisplatin-induced AKI and ferroptosis	(59)
Cisplatin - induced AKI	HK-2	METTL3	Upregulated	SREBP1c	METTL3-mediated upregulation of SREBP1c disrupts mitochondrial energy metabolism by transcriptionally repressing YME1L1, promoting AKI and its progression to CKD	(75)
Cisplatin - induced AKI	HK-2	IGF2BP3	Downregulated	NCBP1	IGF2BP3 inhibits TEC senescence and is involved in cisplatin-induced AKI and its progression to CKD	(62)
Cisplatin - induced AKI	HK2	ZC3H13	Upregulated	NABP1	ZC3H13 knockdown alleviates G2/M cell cycle arrest, apoptosis, and renal injury by affecting NABP1 expression	(51)
Cisplatin - induced AKI	HK2	, FTO	Downregulated	p53	Reduced renal FTO expression increases RNA m6A levels and exacerbates kidney injury in cisplatin-induced AKI	(76)
TNF-α-induced AKI	HK-2	FTO	—	AQP3	FTO alleviates TNF-α-induced TEC injury by targeting AQP3 in an m6A-dependent manner	(77)
Folic acid-induced AKI	TCMK-1	METTL3	Upregulated	ASC	METTL3 promotes inflammatory cytokine release and pyroptosis	(78)
Folic acid-induced AKI	TCMK-1	METTL3,GF2BP3	Upregulated	Hmox1/HO-1	METTL3 mediates m6A modification of HO-1 mRNA and maintains its stability in an IGF2BP3-dependent manner, thereby promoting ferroptosis	(79)

FTO and ALKBH5 are the two known m6A demethylases, both involved in maintaining the dynamic balance of RNA methylation in the normal kidney. In sepsis-associated AKI (56) and I/R-induced AKI (57), FTO is significantly downregulated in human renal tissue, and its overexpression alleviates renal injury. In contrast, ALKBH5 is upregulated in I/R-induced AKI (58) and cisplatin-induced AKI (59) mouse models, and ALKBH5 knockout ameliorates renal dysfunction.

The m6A reader family is diverse, and aberrant expression of YTHDF1, IGF2BP1, IGF2BP2, and IGF2BP3 has been reported in AKI. In AKI, YTHDF1 is upregulated in TECs, where it promotes stress granule assembly by recruiting m6A-modified transcripts and protects the translation of cell survival genes such as *SPHK1*, thereby reducing acute tubular injury (60). In a CLP-induced mouse AKI model, IGF2BP1 enhances MIF expression and activates the NLRP3 inflammasome by stabilizing E2F1 mRNA, thereby driving pyroptosis (61). IGF2BP2 synergizes with METTL3 to stabilize TAB3 mRNA and promote inflammatory signal transduction (50). IGF2BP3 alleviates the senescence of TECs in AKI (62).

In summary, m6A modification exerts multi-level regulatory control over renal physiology and AKI pathology. The dynamic balance between pro-injury and pro-protective factors determines the damage direction and repair outcome of TECs. This regulatory network provides potential targets for epigenetic intervention in AKI.

## 5 The role of m6A modification in acute kidney injury

Emerging evidence indicates that dysregulation of m6A modifications influences key cellular processes, including inflammation, apoptosis, pyroptosis, ferroptosis, autophagy, mitochondrial damage, cellular senescence, and renal fibrosis, thereby emerging as a key contributor to the pathogenesis of AKI. The following section details the mechanisms by which m6A influences the outcome of AKI through these cellular processes. Notably, in some studies, m6A modification regulates multiple cellular processes that are interrelated and mutually reinforcing; the present review focuses on the primary cellular processes affected.

### 5.1 m6A modification regulates inflammation

**Figure 2:**
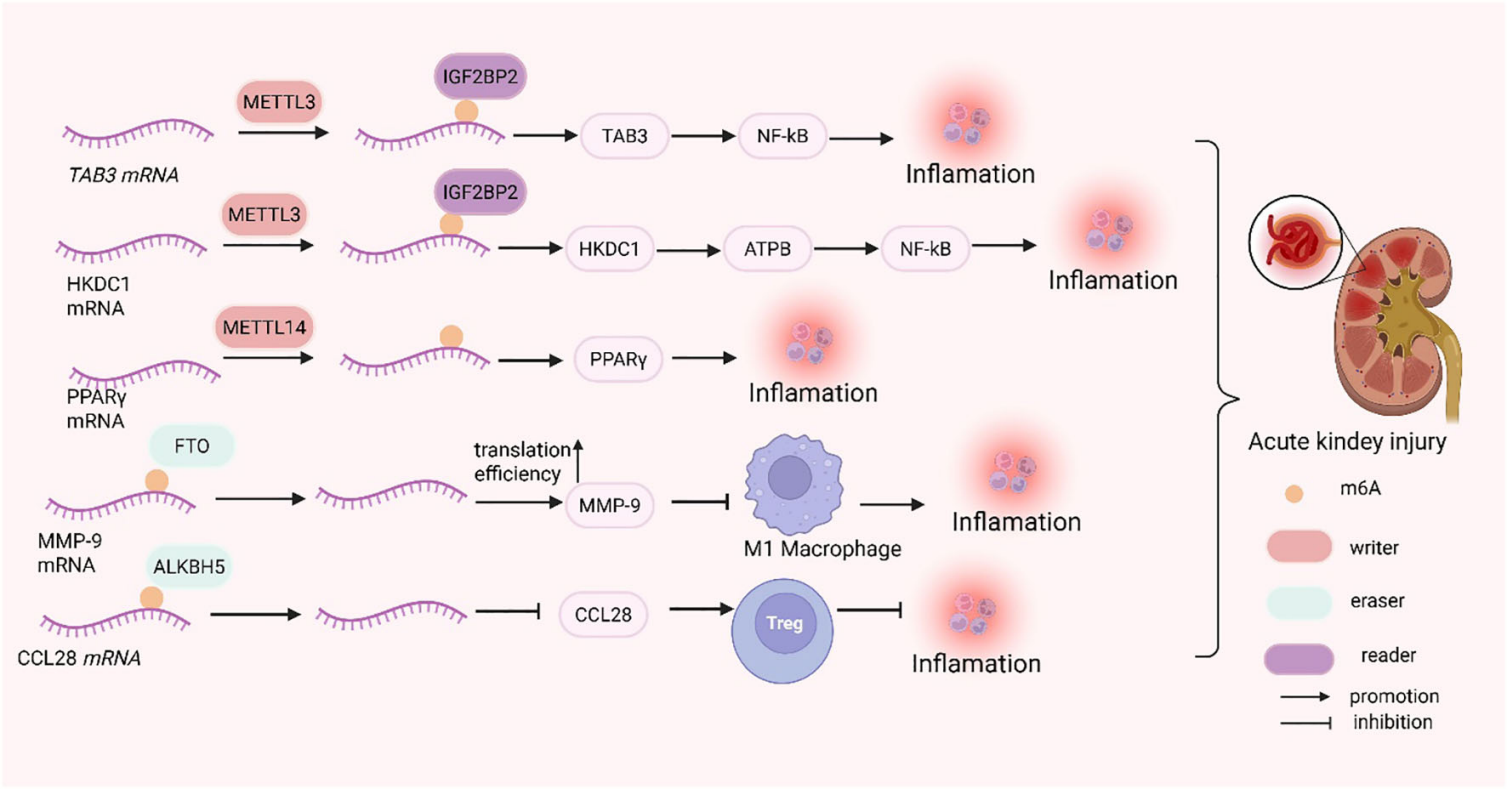
Molecular mechanisms of m6A modification in regulating inflammation during AKI. This schematic illustrates how m6A writers (METTL3 and METTL14) and erasers (FTO and ALKBH5) modulate inflammatory pathways to influence AKI progression. METTL3 and METTL14 mediate m6A methylation of downstream target genes, enhancing their expression and thereby activating inflammatory signaling cascades that drive AKI progression. In contrast, FTO and ALKBH5 remove m6A modifications from target transcripts and regulate immune cell activity, thereby attenuating or modulating the inflammatory response during AKI progression.

A key feature of AKI is the activation of the immune system and induction of inflammatory responses, particularly in sepsis-induced AKI, where renal inflammation can lead to programmed cell death and oxidative stress. Furthermore, excessive activation of the inflammatory response triggers the release of large quantities of pro-inflammatory cytokines, thereby causing a “cytokine storm” that results in damage to the kidneys and other organ systems throughout the body. Consequently, targeting inflammation remains a fundamental therapeutic strategy in the management of AKI. Emerging evidence suggests that m6A modifications are involved in regulating inflammatory responses (**Figure 2**) (80). For example, loss of METTL3 reduces m6A methylation of Traf6, leading to inactivation of the NF-κB and mitogen-activated protein kinase (MAPK) signaling pathways (81).

In various AKI models, METTL3 expression is significantly upregulated, a process that depends on transcriptional activation via binding of c-Jun to the METTL3 promoter region. Overexpression of METTL3 promotes the production of inflammatory cytokines and induces apoptosis, whereas knockdown of METTL3 inhibits the phosphorylation of p65 NF-κB, reduces the production of pro-inflammatory cytokines, and alleviates inflammation and injury in AKI mice, highlighting the crucial role of METTL3-mediated inflammatory responses in AKI (50). Mechanistically, METTL3 mediates m6A modification of *TAB3* mRNA; IGF2BP2 binds to the m6A-modified stop codon region of the mRNA, thereby enhancing its stability and promoting its expression, whereas overexpression of TAB3 further promotes inflammatory responses (50). Similarly, in mice treated with lead acetate, METTL3 expression and m6A levels were significantly upregulated; genetic knockout of METTL3 alleviated renal injury and the inflammatory response induced by lead acetate (63). Mechanistically, METTL3 mediates m6A modification of *HKDC1* mRNA and enhances the stability of *HKDC1* mRNA in an IGF2BP2-dependent manner, thereby promoting its expression. HKDC1 binds to ATPB and antagonizes the ubiquitin ligase MURF1, thereby promoting increased ATPB expression and activating the NF-κB signaling pathway, which in turn promotes renal inflammation (63). Overall, METTL3 is upregulated in AKI and promotes inflammatory responses; both genetic and pharmacological inhibition of METTL3 alleviates renal injury and inflammation, suggesting that METTL3 may be a viable therapeutic target for AKI. Furthermore, studies have reported that METTL14 overexpression promotes PPARγ expression via an IGF2BP2-dependent mechanism, thereby reducing the renal inflammatory response following renal I/R injury in mice (55).

As a vital component of the innate immune system, macrophage activation plays a key role in regulating inflammatory responses. Advances in epigenetics have revealed complex regulatory networks governing macrophage behavior (82). Multiple studies have demonstrated that FTO overexpression protects mice from sepsis-induced AKI. Chen et al. reported that in mice with CLP-induced AKI, FTO expression was reduced in renal tissue and peritoneal macrophages, whereas m6A modification levels were elevated. Conversely, upregulation of FTO inhibited M1 macrophage polarization and the secretion of inflammatory cytokines, thereby alleviating the inflammatory response and AKI. Mechanistically, FTO promotes the translational efficiency of matrix metalloproteinase 9 (MMP-9) mRNA in an m6A-dependent manner, thereby enhancing MMP-9 expression; increased MMP-9 levels contribute to both the resolution of the inflammatory response and the alleviation of renal injury (65). Furthermore, FTO inhibits *SNHG14*, thereby suppressing the NF-κB signaling pathway, significantly reducing the production of inflammatory cytokines such as TNF-α, IL-6, and IL-1β, and alleviating sepsis-induced AKI (56).

Regulatory T cells (Tregs) are important immunosuppressive cells that play a key protective role in tissue repair by suppressing excessive inflammation; their recruitment to the site of injury is partially regulated by chemokines. Additionally, the recruitment of Tregs is regulated by m6A modifications. Chen et al. reported that ALKBH5 is upregulated in mice with I/R-induced AKI; *Alkbh5* knockout mice exhibited milder pathological damage and better renal function following I/R, whereas *Alkbh5* knock-in mice exhibited the opposite results (58). Mechanistically, ALKBH5 binds to and erases m6A modifications on *CCL28* mRNA, thereby reducing the binding of IGF2BP3 to m6A-modified sites on *CCL28* mRNA. This leads to decreased *CCL28* mRNA stability and reduced expression; subsequent downregulation of CCL28 inhibits the recruitment of renal Tregs, increases macrophage and neutrophil levels, and ultimately promotes inflammation and AKI. Conversely, *Alkbh5* deficiency increases CCL28-mediated Treg recruitment, suppresses inflammatory cell infiltration, and ultimately attenuates AKI (58).

In summary, in AKI, the expression of m6A regulatory elements is dysregulated, characterized by upregulation of the m6A “writer” METTL3, downregulation of the m6A “eraser” FTO, and consequently increased levels of m6A modification. This leads to dysregulation of downstream target gene expression, which in turn exacerbates inflammation and AKI through mechanisms such as activation of inflammatory pathways, promotion of cytokine release, M1 macrophage polarization, and suppression of Treg cell recruitment.

### 5.2 m6A modification regulates pyroptosis

A growing body of evidence has established pyroptosis as a critical pathogenic mechanism in AKI. Concurrently, recent studies have demonstrated that m6A modification plays a pivotal role in the regulation of pyroptosis (83, 84). For instance, knockdown of METTL3 inhibits NLRP3 inflammasome activation and downregulates pyroptosis-related transcripts, thereby suppressing pyroptosis (83).

**Figure 3:**
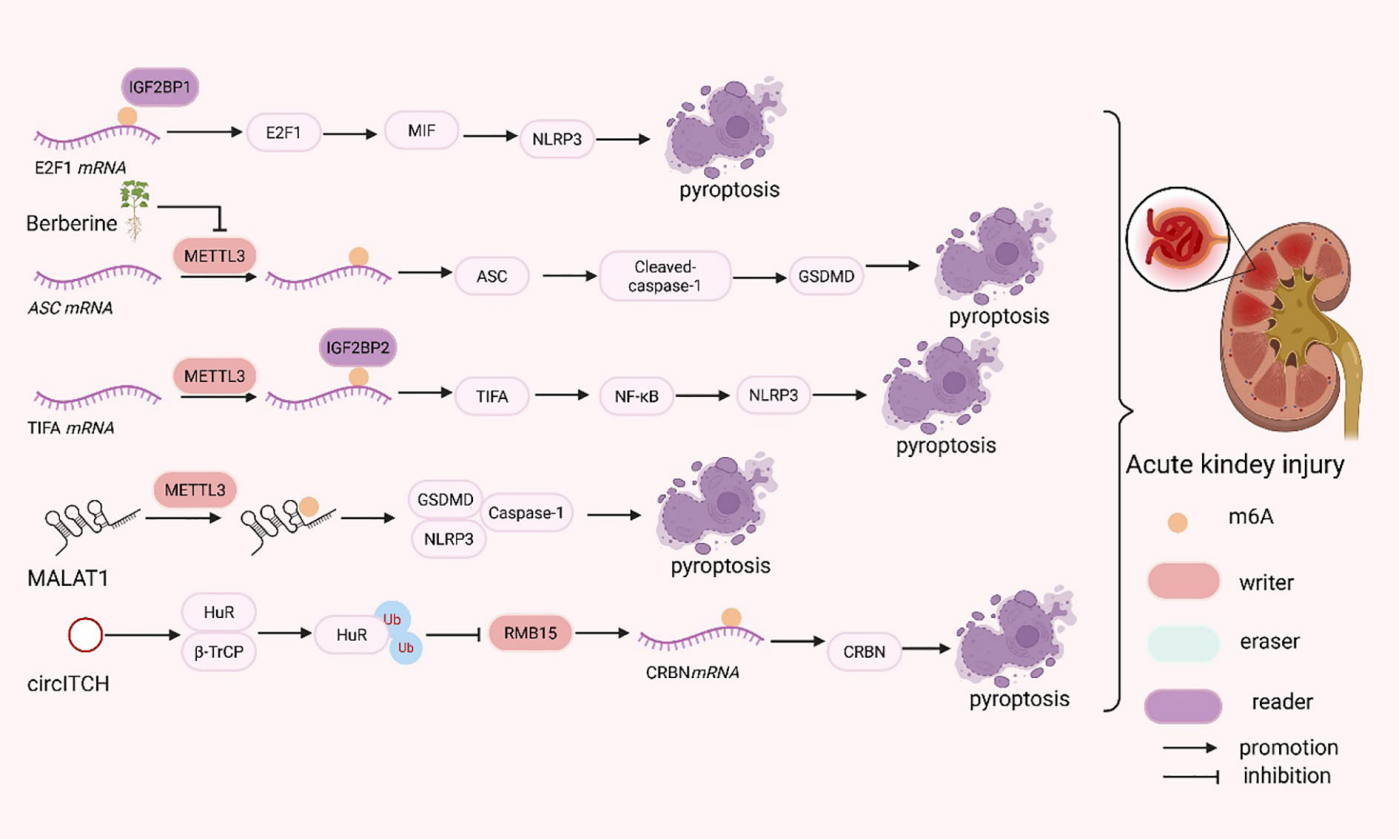
Molecular mechanisms of m6A modification in regulating pyroptosis during AKI. m6A modification regulates the expression of key target genes—including *E2F1, ASC, TIFA, MALAT1*, and *CRBN*—thereby modulating pyroptosis and influencing the onset and progression of AKI.

Emerging evidence has increasingly implicated m6A modification in the regulation of pyroptosis during AKI (**Figure 3**). In AKI, upregulation of m6A-related proteins correlates with the occurrence of pyroptosis, suggesting that both m6A modification and pyroptosis play critical roles in AKI. Mao et al. reported that in a CLP-induced mouse model of AKI, elevated m6A levels and upregulated IGF2BP1 expression in TECs were accompanied by increased levels of the inflammatory cytokines IL-6 and TNF-α, as well as the occurrence of pyroptosis; knockdown of IGF2BP1 attenuated pyroptosis, improved renal function, indicating that IGF2BP1 is a key factor in inducing pyroptosis and promoting the progression of AKI (61). Mechanistically, IGF2BP1 recognizes m6A-modified sites on E2F1 and upregulates E2F1 expression; E2F1, as a transcription factor, promotes MIF expression. MIF is a component of the NLRP3 inflammasome and is crucial for NLRP3 activation, which ultimately induces pyroptosis (61).

METTL3 plays a significant role in the progression of AKI through regulating pyroptosis. Li et al. reported that in a folic acid -induced AKI mouse model, METTL3 expression levels were significantly correlated with pyroptosis and inflammatory responses in TECs. Knockdown of METTL3 reduced ASC protein expression, decreased the release of inflammatory cytokines, inhibited pyroptosis, and alleviated folic acid -induced AKI (78). Mechanistically, METTL3 mediates m6A methylation of *ASC* mRNA and promotes its expression. ASC possesses a CARD domain that interacts with caspases 1, 8, and 9, thereby inducing pyroptosis (78). Another study demonstrated that METTL3 expression was altered in an I/R-induced AKI model. METTL3 silencing inhibited pyroptosis and alleviated I/R-induced renal injury, whereas METTL3 overexpression exacerbated pyroptosis and renal injury. Mechanistically, METTL3 mediates m6A modification of TIFA mRNA; IGF2BP2 recognizes the m6A modification sites on TIFA mRNA to enhance its stability and expression. TIFA further promotes NLRP3 transcription via the NF-κB signaling pathway, thereby driving pyroptosis and promoting the progression of AKI (74). Furthermore, m6A modification can regulate pyroptosis in AKI by modulating the expression of lncRNAs. Studies have shown that the lncRNA MALAT1 is a key factor in promoting renal inflammation and pyroptosis in AKI; its expression is significantly upregulated during LPS-induced injury (69). Knockdown of MALAT1 significantly reduces inflammatory cytokine levels and pyroptosis in TECs. Bioinformatics analysis revealed that METTL3 interacts with MALAT1 and helps regulate its expression. Knockdown of METTL3 reduced the expression of pyroptosis markers and inflammatory cytokines, whereas MALAT1 overexpression partially reversed these effects. Mechanistically, METTL3 directly binds to MALAT1, enhancing its m6A modification and promoting its expression, thereby facilitating pyroptosis and the progression of AKI (69).

A study reported that adipose-derived stem cell-derived exosomes (ADSCs-EVs) carrying circITCH can reduce creatinine and blood urea nitrogen levels, alleviate tubular damage, and inhibit pyroptosis in renal tissues and cells. Mechanistically, ADSCs-EVs deliver circITCH into cells, upregulating its expression, enhancing the interaction between HuR and β-TrCP, promoting HuR ubiquitination, and weakening the interaction between HuR and RBM15 mRNA. This reduces m6A modification on CRBN, thereby inhibiting IGF2BP1-mediated CRBN expression, reducing pyroptosis in TECs, and ultimately alleviating LPS-induced AKI (70).

In summary, pyroptosis plays a crucial role in the initiation and progression of AKI, and because pyroptosis is regulated by m6A modification, targeting m6A modification to inhibit pyroptosis may represent a promising therapeutic strategy for AKI.

### 5.3 m6A-mediated regulation of ferroptosis

**Figure 4:**
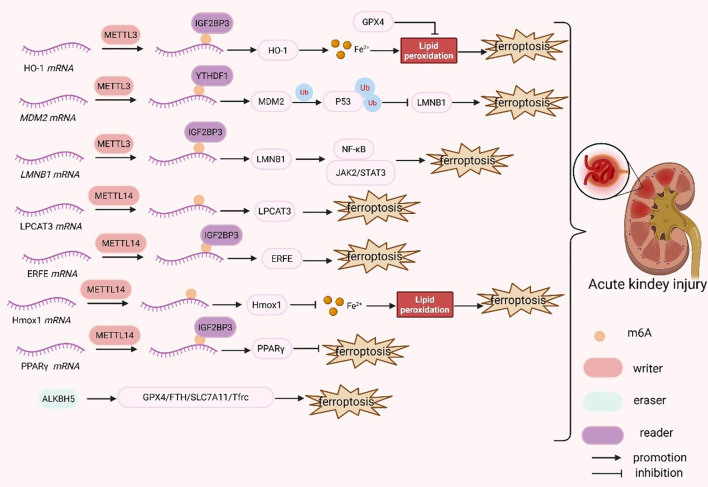
Molecular mechanisms of m6A modification in regulating ferroptosis during AKI. m6A modification regulates the expression of key ferroptosis-related target genes—including *HO-1, MDM2, LMNB1, LPCAT3, ERFE*, and *PPARγ*—thereby modulating ferroptosis and influencing the onset and progression of AKI.

Since the discovery of ferroptosis, its role in the development of AKI and the subsequent progression from AKI to CKD has been extensively studied (24, 25). Furthermore, a growing body of research indicates that m6A modification, as a key regulator of ferroptosis, plays a crucial role in both ferroptosis and the progression of AKI (**Figure 4**) (85). A bioinformatics analysis revealed that the expression levels of ferroptosis-associated genes (SAT1, ACSL4, and NFE2L2) and m6A methylation-associated genes (YTHDF3, WTAP, and IGF2BP3) were significantly elevated and positively correlated in AKI patients (86).

A growing body of research indicates that METTL3 plays a crucial role in both ferroptosis and AKI. Lv et al. (79) reported that METTL3 expression is upregulated and m6A modification levels are elevated in AKI patients and folic acid -induced AKI mice. Furthermore, in folic acid -induced AKI mice, NGAL expression and ferric iron levels increased, whereas E-cadherin and GPX4 expression decreased and GSH levels fell, indicating that the development of AKI is closely associated with iron overload-induced ferroptosis. Additionally, knockdown or pharmacological inhibition of METTL3 alleviated damage and ferroptosis in TECs both *in vivo* and *in vitro*, thereby preserving renal function. Mechanistically, METTL3 mediates m6A modification of heme oxygenase 1 (Hmox1/HO-1) mRNA; IGF2BP3 acts as an m6A “reader” that binds to m6A methylation sites on Hmox1 mRNA to maintain its stability and promote its expression (79). Hmox1/HO-1 is an inducible stress protein involved in heme catabolism. Under specific pathological conditions, overexpression of Hmox1/HO-1 can catalyze the release of free iron, promote lipid peroxidation, and thereby drive ferroptosis (87, 88). Furthermore, Hu et al. reported that in LPS-treated HK-2 cells and CLP-induced AKI mouse models, upregulation of METTL3, MDM2, and LMNB1 and downregulation of p53 were observed, accompanied by ferroptosis and mitochondrial damage (66). Following simultaneous knockdown of METTL3 and YTHDF1, the levels of m6A methylation of MDM2 mRNA and MDM2 protein decreased, thereby inhibiting mitochondrial damage and ferroptosis and alleviating AKI. Mechanistically, METTL3 mediates m6A modification of MDM2 mRNA, whereas YTHDF1 binds to MDM2 mRNA in an m6A modification-dependent manner and promotes MDM2 expression (66). As an E3 ubiquitin ligase, MDM2 can ubiquitinate p53, thereby triggering its degradation to enhance LMNB1 expression, induce ferroptosis, and exacerbate renal injury (66, 89). Another study also confirmed that LMNB1 is regulated by WTAP-mediated m6A methylation; in a CLP-induced AKI mouse model, both WTAP and LMNB1 expression were upregulated. Knockdown of LMNB1 or WTAP promoted HK-2 cell survival and inhibited apoptosis, inflammation, mitochondrial damage, and ferroptosis, whereas LMNB1 overexpression reversed the effects of WTAP knockdown. Mechanistically, WTAP was shown to promote LMNB1 expression via m6A methylation, which in turn activates the NF-κB and JAK2/STAT3 pathways, thereby promoting cellular inflammation, mitochondrial damage, and ferroptosis (71).

As an important methyltransferase, METTL14 plays a significant role in both ferroptosis and the development of AKI. Studies have reported that in LPS-treated TCMK-1 cells, METTL14 expression, m6A levels, and ferroptosis are significantly elevated. Knockdown of METTL14 reduces cell viability, malondialdehyde (MDA) levels, Fe²^+^ concentration, and lipid peroxidation, whereas increasing GSH levels, thereby ameliorating AKI in sepsis-induced mice. Furthermore, LPCAT3 overexpression can antagonize the inhibitory effect of METTL14 downregulation on ferroptosis (52). Mechanistically, METTL14 enhances the stability of LPCAT3 through m6A modification, thereby promoting LPCAT3 expression and inducing ferroptosis (52). Furthermore, studies have reported that in cisplatin-treated HK-2 cells, the expression of METTL14, IGF2BP3, and ERFE is upregulated, accompanied by increased m6A modification levels and ferroptosis (53). ERFE is a hormone secreted by erythrocytes that acts as a major regulator of iron homeostasis by inhibiting hepcidin production in the liver; pathological overexpression of ERFE induces iron overload, oxidative stress, and ferroptosis (90, 91). Following METTL14 knockdown, ERFE expression and ferroptosis are suppressed, whereas METTL14 overexpression produces the opposite effect. Furthermore, overexpression of ERFE reversed the protective effects of METTL14 knockdown, demonstrating that ERFE is a key downstream effector of METTL14. Mechanistically, METTL14 directly binds to and increases the m6A levels of ERFE mRNA; IGF2BP3 acts as a “reader” by directly binding to m6A-modified ERFE mRNA, thereby enhancing ERFE mRNA stability and expression, which in turn induces cellular ferroptosis (53).

However, other studies have also shown that METTL14 can suppress ferroptosis and alleviate renal injury in mice with AKI. Yang et al. reported that METTL14 is significantly downregulated in tissues from human patients with sepsis-associated AKI and in the mouse CLP model. Its overexpression improved renal function, reduced tissue damage and mortality, and suppressed the production of pro-inflammatory cytokines. Furthermore, knockdown of METTL14 significantly upregulates Hmox1 expression, exacerbating ferroptosis and renal injury (54). Mechanistically, Hmox1 mRNA is a direct target of METTL14; its m6A modification levels increase upon METTL14 overexpression, which promotes its m6A-dependent degradation, thereby limiting iron accumulation and lipid peroxidation (54). Furthermore, Liu et al. reported that in an I/R-induced AKI model, knockdown of METTL14 significantly reduced the m6A levels and expression of PPARγ mRNA in TECs (55). PPARγ is a nuclear receptor for which activation has been found to alleviate renal injury and improve renal function, whereas dysregulation of PPARγ may render TECs more susceptible to ferroptosis, thereby exacerbating renal injury (92, 93). Furthermore, METTL14 overexpression promoted PPARγ expression and inhibited ferroptosis; knockdown of PPARγ partially reversed the inhibitory effect of METTL14 overexpression on ferroptosis in TECs, indicating that PPARγ expression and ferroptosis are regulated by METTL14 (55). Mechanistically, METTL14 mediates m6A methylation at the MUT4 site on PPARγ mRNA; IGF2BP2 regulates the stability of PPARγ mRNA in TECs via an m6A-dependent mechanism, thereby promoting PPARγ expression, inhibiting ferroptosis in TECs, and alleviating renal injury in I/R mice (55). Current evidence suggests that METTL14 exhibits a dual role in the onset and progression of AKI, likely mediated by multiple mechanisms.First, the target genes of METTL14 include both ferroptosis-inhibiting and ferroptosis-promoting factors. Consequently, depending on which target genes are predominantly affected, METTL14 can either promote or inhibit AKI progression.Second, m6A “readers” possess diverse functions. Depending on the specific reader involved, m6A modification can either enhance the stability and expression of target genes or promote their degradation and translational repression. This functional diversity represents another important source of METTL14’s dualistic role in AKI.Additionally, differences in AKI models, variations in detection time points, and discrepancies in experimental techniques may also contribute to the observed differences in METTL14’s functional outcomes across studies.

ALKBH5, an important demethylase, is upregulated in cisplatin-induced AKI mice; Alkbh5 knockout was found to alleviate cisplatin-induced renal dysfunction, whereas its knock-in exacerbated the effects. Furthermore, in cisplatin-treated mice, the levels of GPX4, FTH, and SLC7A11 proteins decreased, whereas Tfrc expression increased significantly. Notably, the expression levels of GPX4, FTH, SLC7A11, and Tfrc were reversed in Alkbh5 knockout mice, suggesting that Alkbh5 knockdown significantly reduced cisplatin-induced ferroptosis (59). Given that ALKBH5 acts as an m6A demethylase, it may exacerbate cisplatin-induced ferroptosis and tubular damage in TECs by removing m6A modifications from the mRNA of its target genes, thereby upregulating the expression of key ferroptosis-promoting proteins. However, the study did not further investigate the levels of m6A modification or identify potential target genes.

### 5.4 m6A modification regulates apoptosis

**Figure 5:**
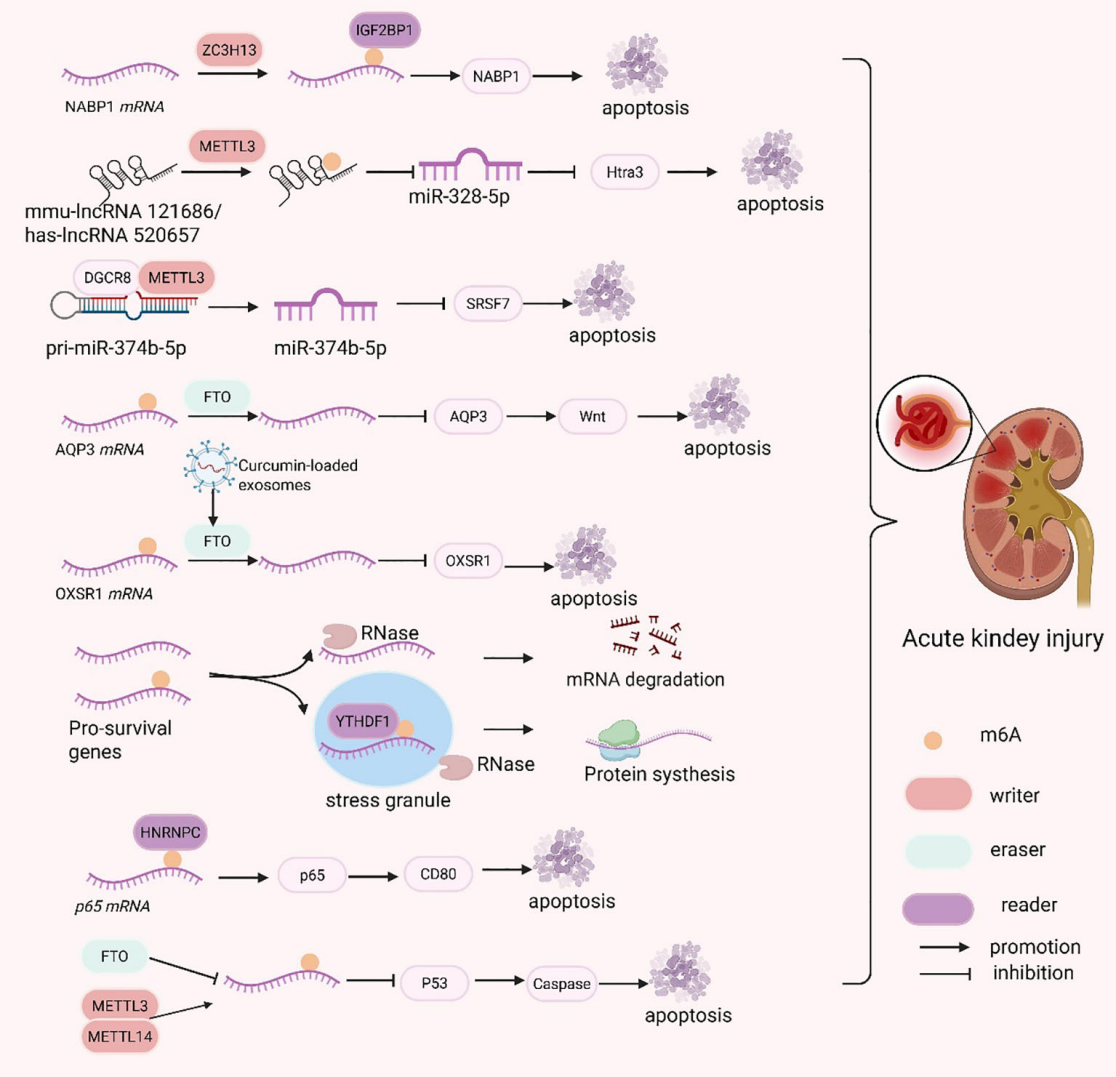
Molecular mechanisms of m6A modification in regulating apoptosis during AKI. m6A modification regulates the expression of key apoptosis-related targets—including *NABP1*, the long non-coding RNAs mmu-lncRNA 121686 and has-lncRNA 520657, miR-374b-5p, *AQP3*, *OXSR1*, *p65*, and *p53*—thereby modulating apoptotic cell death and influencing AKI progression.

Apoptosis is a form of programmed cell death; although this process is essential for maintaining organismal health, dysregulation of cell death resulting from excessive or defective apoptosis has been linked to a variety of disease states. A growing body of experimental evidence supports the notion that TECs are highly susceptible to apoptosis upon stimulation by various noxious factors, a process that plays a crucial role in the pathogenesis of AKI (14). Furthermore, apoptosis is also regulated by m6A modifications (**Figure 5**).

Studies have shown that METTL3 regulates the expression of non-coding RNAs in an m6A modification-dependent manner, thereby inducing apoptosis in AKI. Pan et al. reported that in I/R-induced AKI mouse and cell models, the expression of METTL3, mmu-lncRNA 121686, and its homologue has-lncRNA 520657 was upregulated, whereas Mettl3 knockout significantly alleviated damage and apoptosis in TECs and improved renal function (64). Mechanistically, METTL3 mediates m6A modification of mmu-lncRNA 121686 and has-lncRNA 520657, thereby promoting their upregulation; these lncRNAs attenuate the inhibitory effect of miR-328-5p on its downstream target gene Htra3, thereby promoting Htra3 expression, which ultimately contributes to TECs apoptosis (64). Furthermore, in an I/R-induced AKI model, miR-374b-5p levels were significantly upregulated; by inhibiting the expression of its downstream target gene SRSF7, miR-374b-5p suppressed cell viability and exacerbated apoptosis, thereby promoting I/R -induced damage to TECs (73). Further studies have shown that METTL3 interacts with the microprocessor protein DGCR8 and regulates the maturation of pri-miR-374b-5p in an m6A-dependent manner, downregulating SRSF7 expression and thereby promoting the development of AKI (73). Furthermore, the methyltransferase ZC3H13 also promotes cell apoptosis and exacerbates AKI through an m6A modification-dependent mechanism. Sheng et al. (51) reported that in cisplatin-induced AKI mouse and cell models, m6A modification level and ZC3H13 expression were significantly upregulated. Functionally, ZC3H13 induces G2/M cell cycle arrest and apoptosis via the p53/p21 signaling pathway, ultimately leading to renal injury. Conversely, knockdown of ZC3H13 alleviated G2/M cell cycle arrest and apoptosis and mitigated renal injury. Mechanistically, ZC3H13 mediates m6A modification of nucleic acid-binding protein 1 (NABP1) mRNA; IGF2BP1 recognizes and binds to NABP1 mRNA in an m6A modification-dependent manner, enhancing its stability, thereby promoting apoptosis and renal injury (51).

FTO is frequently downregulated in AKI, whereas its upregulation can inhibit apoptosis and alleviate AKI. Li et al. reported that TNF-α treatment significantly promoted apoptosis in TECs. Overexpression of FTO attenuated TNF-α-induced apoptosis and promoted cell survival, whereas knockdown of FTO further exacerbated the effects of TNF-α (77). Mechanistically, FTO targets AQP3 via an m6A-dependent mechanism, thereby compromising AQP3 stability and further attenuating AQP3’s inhibition of the Wnt pathway, thus mitigating TNF-α-induced damage to TECs (77). In cisplatin-induced AKI, FTO expression is reduced, and m6A levels are elevated. Inhibition of FTO further elevated m6A levels and promoted apoptosis in TECs; consistently, overexpression of METTL3 and METTL14 increased m6A levels and promoted apoptosis (94). Furthermore, FTO overexpression reduced the mRNA levels of p53, a key regulator of apoptosis, by 36%, whereas FTO knockdown increased p53 mRNA levels by 20.4-fold, suggesting that FTO-mediated inhibition of p53 expression may underlie its anti-apoptotic effect (94). Another study reported that FTO suppresses LPS-induced apoptosis and damage in HK2 cells by inhibiting OXSR1 expression through m6A demethylation (76). In summary, these studies indicate that in AKI, renal FTO expression is reduced, thereby increasing m6A levels and promoting cell apoptosis as well as the development of AKI. Consequently, FTO overexpression may represent a viable therapeutic strategy for AKI.

HNRNPC is an m6A-dependent RNA-binding protein that promotes apoptosis and plays a significant role in the development of AKI. Chen et al. reported that in a sepsis-associated AKI model, total m6A levels and HNRNPC expression were significantly upregulated; following knockdown of HNRNPC, apoptosis was significantly inhibited, levels of inflammatory cytokines were downregulated, and renal function improved. Mechanistically, METTL3-mediated m6A RNA methylation is crucial for HNRNPC-induced apoptosis. HNRNPC binds to m6A sites on NF-κB p65 mRNA and enhances its stability; NF-κB, as a transcription factor, promotes CD80 expression, thereby inducing apoptosis and exacerbating AKI (67). Furthermore, another important ‘reader’ protein, YHDF1, promotes the formation of stress granules (SGs), which play a crucial role in protecting renal tubules from stress-induced insults. SGs are membraneless organelles formed by cells under stress conditions, serving to temporarily store, suppress translation of, and protect specific mRNAs and proteins, thereby helping cells survive stressful conditions (95). Studies have shown that under stress conditions, m6A-modified mRNAs accumulate significantly in SGs within TECs, thereby protecting the cells from apoptosis. The formation of SGs depends on the function of YTHDF1; knockout of Ythdf1 in renal tubular cells leads to a significant reduction in the accumulation of m6A-modified mRNA within SGs, accompanied by increased cell apoptosis under stress conditions, whereas Ythdf1 knockout mice exhibit more severe AKI (60). Mechanistically, YTHDF1 selectively recruits m6A-modified mRNAs into SGs. Through this mechanism, these m6A-modified mRNAs, which promote cell survival, can evade degradation following stress exposure and maintain transcript abundance. SPHK1 mRNA, representing a gene that promotes cell proliferation and survival, is one of the m6A-modified mRNAs protected by YTHDF1 under stress conditions. Depletion of YTHDF1 leads to a decrease in SPHK1 levels and increased cell apoptosis (60). In this study, YTHDF1 does not function as a classic post-transcriptional regulator but rather alters the spatial distribution of mRNA to prevent degradation; this represents a significant breakthrough in our understanding of the “reader” function of m6A.

### 5.5 m6A modification regulates autophagy

**Figure 6:**
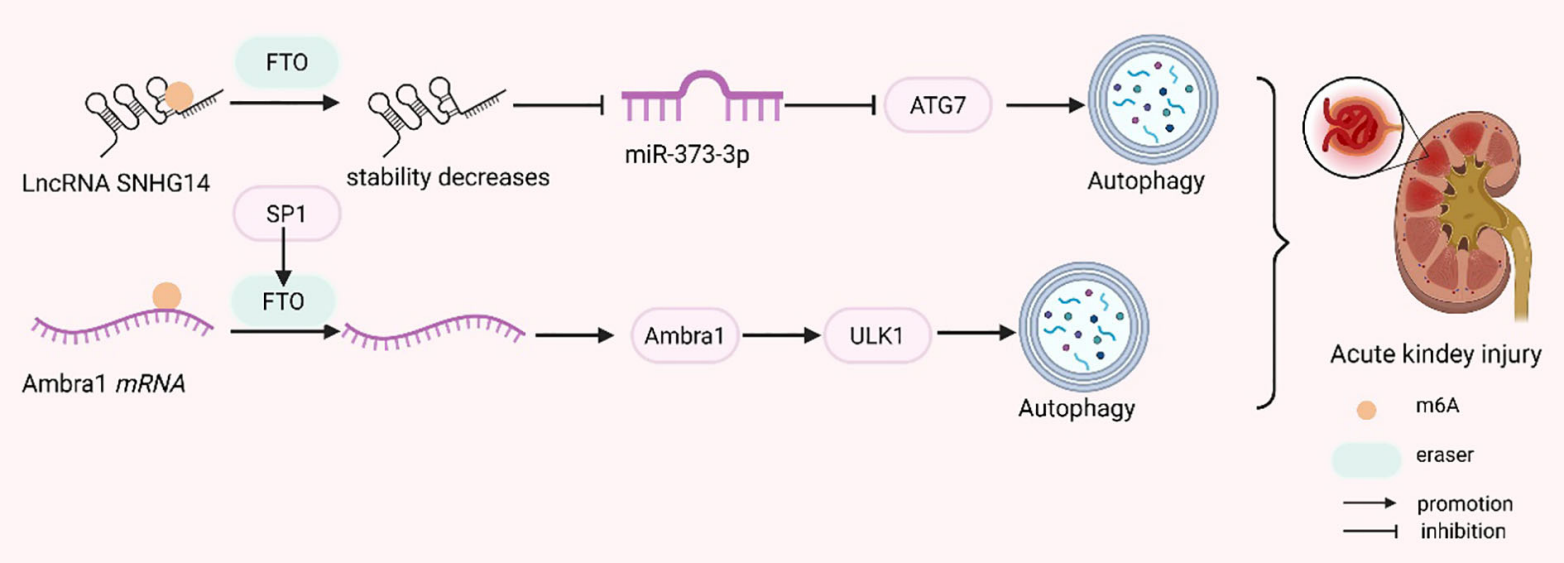
Molecular mechanisms of m6A modification in regulating autophagy during AKI. m6A modification regulates the expression of key autophagy-related targets, including the long non-coding RNA *SNHG14* and the autophagy regulator *AMBRA1*, thereby modulating autophagic flux and influencing AKI progression.

As an intracellular defense mechanism, autophagy enables cells to cope with stressful environments by clearing damaged organellar proteins and recycling them to provide energy and nutrients; through this function, it plays a significant role in maintaining cellular homeostasis (96, 97). Similarly, autophagy is one of the mechanisms by which the kidneys mount adaptive responses to various stressors. Dysregulated autophagy may also contribute to the development of various kidney diseases (26). As a key regulator of autophagy, m6A modification plays a significant role in the pathogenesis of AKI (**Figure 6**). Yang et al. (56) reported that in patients with sepsis-associated AKI and in mouse models of AKI, FTO expression was downregulated whereas m6A methylation levels were upregulated. Furthermore, FTO overexpression inhibited LPS-induced apoptosis and autophagy in HK-2 cells, thereby ameliorating AKI. Mechanistically, the lncRNA SNHG14 acts as a target of FTO. Overexpression of FTO removes the m6A modification from SNHG14, thereby reducing its stability and inhibiting its expression. This, in turn, enhances the suppression of its downstream target ATG7 by miR-373-3p, ultimately inhibiting cellular autophagy, protecting TECs, and ameliorating the progression of AKI (56).

However, another study suggests that FTO ameliorates I/R-induced AKI by inducing autophagy. Chen et al. reported that FTO expression was downregulated in I/R-induced rat kidney tissue. Conversely, FTO overexpression ameliorated renal injury in I/R rats. Notably, FTO overexpression significantly increased the levels of the autophagy-associated proteins LC3 and ULK1. When autophagy was inhibited, the protective effect of FTO in AKI was attenuated, suggesting that FTO amelioration of AKI depends on the activation of autophagy (57). Mechanistically, FTO removes the m6A modification from Ambra1 mRNA, thereby promoting its expression (57). AMBRA1 interacts with the E3 ubiquitin ligases TRIM32 and TRAF6 to promote the ubiquitination of ULK1, a key regulator of autophagy, thereby enhancing its protein stability. This, in turn, promotes autophagy and ameliorates I/R-induced AKI (98, 99). Furthermore, SP1, acting as an upstream transcription factor, directly interacts with the FTO promoter to enhance FTO expression, thereby promoting autophagy via upregulation of the AMBRA1/ULK1 signaling pathway and inhibiting oxidative stress and I/R-induced AKI (57).

In summary, FTO regulates autophagy in an m6A modification-dependent manner, thereby mitigating renal injury. However, in the aforementioned studies, FTO both inhibited and promoted autophagy, respectively, yet ultimately ameliorated the progression of AKI in both cases, indicating that FTO-mediated regulation of autophagy is context-dependent and that the role of autophagy in AKI is similarly context-dependent.

### 5.6 m6A modification regulates mitochondrial function

**Figure 7:**
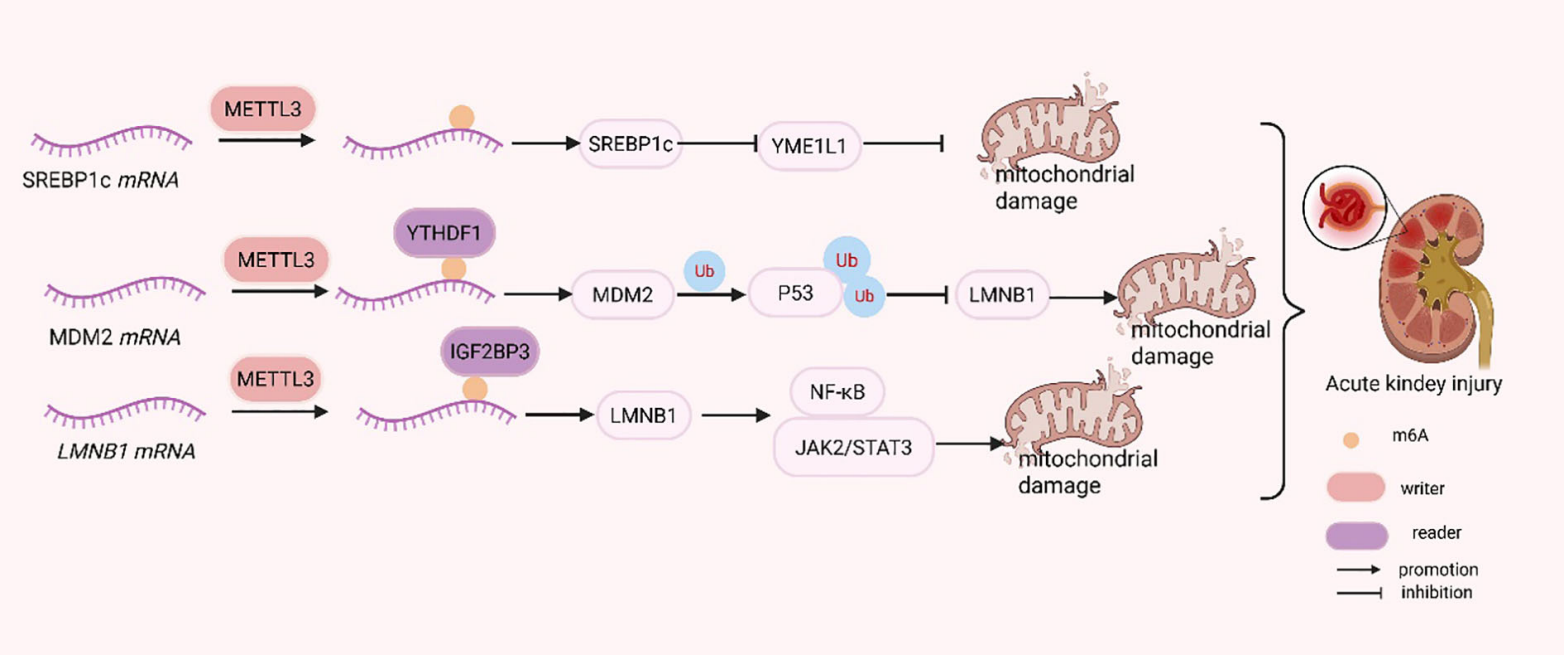
Molecular mechanisms of m6A modification in regulating mitochondrial function during AKI. m6A modification regulates the expression of key mitochondrial function-related targets—including *SREBP1c*, *MDM2*, and *LMNB1*—thereby modulating mitochondrial homeostasis and influencing AKI progression.

The kidney is a highly metabolically active organ that relies on abundant mitochondria to generate energy, which is essential for maintaining normal renal function (100). Mitochondrial damage may directly lead to renal injury, and a growing body of research suggests that mitochondrial dysfunction is one of the most characteristic features and key pathological processes of AKI. Recent studies indicate that during AKI, imbalances in mitochondrial homeostasis and dynamics lead to tubular apoptosis and subsequent interstitial fibrosis (101). Several studies have demonstrated that m6A modification regulates mitochondrial function and plays a significant role in the development of AKI (**Figure 7**). Wang et al. reported that METTL3-mediated m6A modification enhances the stability of SREBP1c mRNA, thereby upregulating its expression. Upregulation of SREBP1c leads to transcriptional repression of YME1L1 by directly binding to the YME1L1 promoter region (75). YME1L1 is an inner mitochondrial membrane protein capable of mediating the remodeling, unfolding, and degradation of mitochondrial proteins, thereby stabilizing mitochondrial structure and maintaining mitochondrial function (102). Consequently, transcriptional repression of YME1L1 disrupts mitochondrial energy metabolism, promoting cisplatin-induced AKI and its progression to CKD (75). Other studies have reported that METTL3 mediates m6A modification of MDM2 mRNA and enhances its expression in a YTHDF1-dependent manner, further promoting mitochondrial damage and the progression of AKI via the MDM2-p53-LMNB1 axis (66). Furthermore, WTAP-mediated m6A modification promotes LMNB1 expression and activates the NF-κB and JAK2/STAT3 pathways, thereby promoting mitochondrial damage in TECs during AKI (71).

Taken together, these findings suggest that m6A modification disrupts mitochondrial energy metabolism, promotes mitochondrial damage, and thus contributes to AKI and its progression to CKD. Targeting the m6A modification axis may ameliorate mitochondrial dysfunction during AKI, offering a potential novel therapeutic strategy for AKI.

### 5.7 m6A modification regulates cellular senescence and renal fibrosis

**Figure 8:**
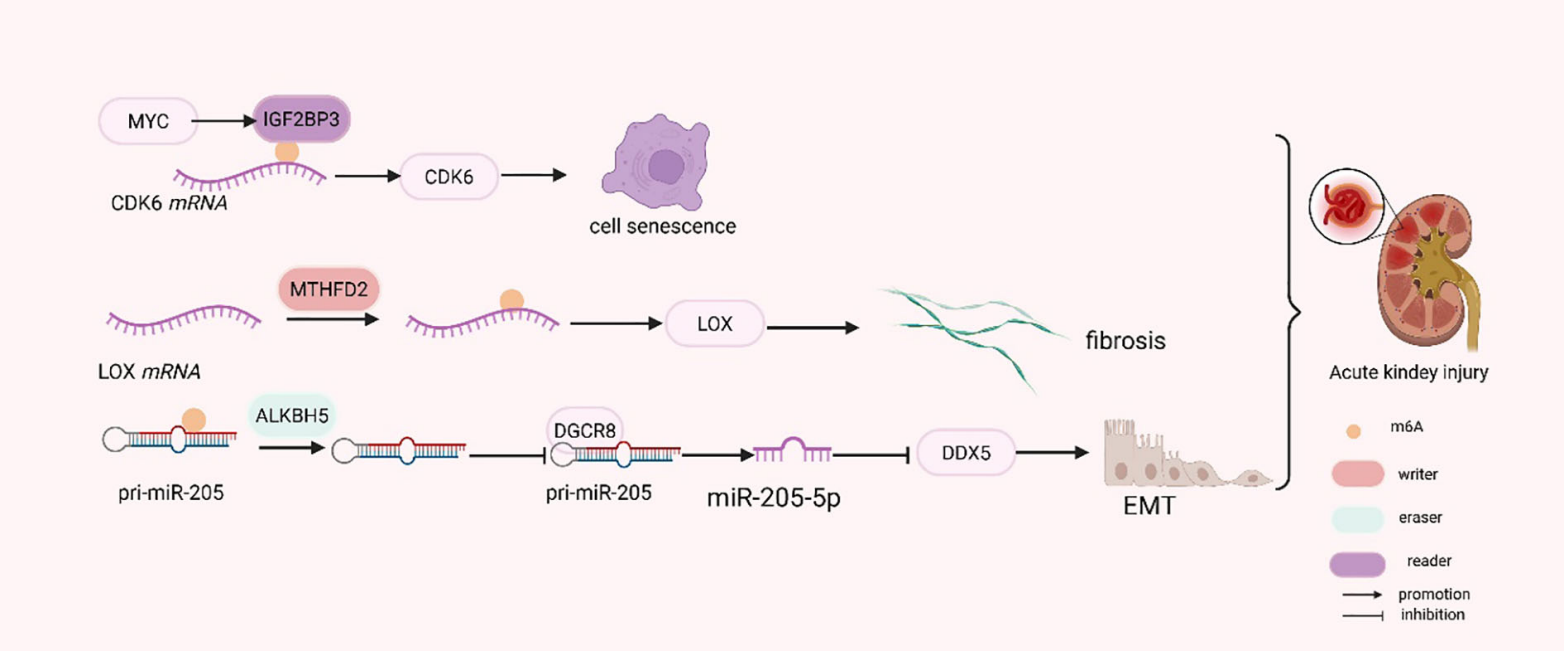
Molecular mechanisms of m6A modification in regulating cellular senescence and renal fibrosis during AKI. m6A modification regulates the expression of key targets involved in senescence and fibrosis—including *CDK6*, *LOX*, and miR-205—thereby modulating cellular senescence and extracellular matrix deposition, and ultimately influencing AKI progression and the transition to CKD.

Cellular senescence is an irreversible process of cell cycle arrest, accompanied by changes at the transcriptional, metabolic, and secretory levels, as well as alterations in cell morphology and chromatin organization (103, 104). To date, animal studies indicate that cellular senescence occurs in the early stages of AKI and is critical for prognosis. Cellular senescence plays a dominant role in the progression of AKI to CKD (105). Furthermore, m6A modification is closely associated with cellular senescence (**Figure 8**) (106, 107). Studies have shown that IGF2BP3 is involved in cisplatin-induced senescence of TECs, whereas IGF2BP3 overexpression alleviates TEC senescence *in vitro (*62). Mechanistically, IGF2BP3 inhibits TEC senescence by recognizing m6A modifications, recruiting nuclear cap-binding protein subunit 1 (NCBP1), and enhancing the stability and expression of CDK6 mRNA. Furthermore, MYC regulates IGF2BP3 transcription by binding to the IGF2BP3 promoter (62).

Renal fibrosis is a pathological process characterized by excessive deposition of extracellular matrix, manifesting as chronic kidney injury and impaired adaptive structural repair. Renal fibrosis, often resulting from the transformation of various renal cells into myofibroblasts, is a key factor in the progression from AKI to CKD (108). Studies have reported that MTHFD2-regulated m6A methylation levels play a significant role in AKI and renal fibrosis. In CLP-induced AKI mouse kidneys, MTHFD2 expression is significantly elevated; conversely, knockdown of MTHFD2 in mouse kidney tissue significantly reduces the m6A modification levels of Lox mRNA and LOX expression, thereby protecting renal function and ameliorating AKI (68). LOX is a member of the lysyl oxidase family, plays a key role in extracellular matrix cross-linking, and is associated with fibrosis (109). Mechanistically, knockdown of MTHFD2 reduces the levels of the methylation donor S-adenosylmethionine (SAM), thereby inhibiting m6A modification of LOX mRNA, which in turn reduces its stability and expression. Reduced LOX expression significantly inhibits collagen accumulation and interstitial fibrosis in mouse kidney tissue (68). The epithelial-mesenchymal transition (EMT) process contributes to renal fibrosis and subsequent acute and chronic kidney injury. Alkbh5 is highly expressed in LPS-induced HK-2 cells; however, inhibition of Alkbh5 enhances cell survival and reduces EMT (72). Mechanistically, ALKBH5 inhibits the binding of DGCR8 to pri-miR-205 by removing m6A modifications, thereby reducing miR-205-5p expression, which in turn upregulates DDX5 expression, promoting EMT following AKI and the subsequent transition to CKD (72).

In summary, m6A modification promotes cellular senescence and renal fibrosis, thereby contributing to the progression of AKI and its transition to CKD. Targeting m6A modification to inhibit cellular senescence and renal fibrosis may represent a viable therapeutic strategy for AKI.

## 6 Strategies for treating AKI based on m6A modification

Given that RNA modifications, particularly m6A modifications, play important biological roles in various types of AKI, the development of m6A-targeting drugs has emerged as a promising strategy. Altered levels of m6A modification have been observed in AKI patients and animal models, and modulating m6A modification has been shown to ameliorate AKI in animal models, further demonstrating the feasibility of targeting m6A modification for the treatment of AKI.

The development of inhibitors and agonists targeting m6A regulators represents a viable therapeutic strategy for AKI; when combined with existing AKI treatments, this approach may enhance therapeutic outcomes. Although these inhibitors and agonists have not yet been widely adopted in clinical practice, they have demonstrated the potential to ameliorate renal injury in animal models of AKI. As the most common and important “writer” of m6A modification, METTL3 is upregulated in various AKI models. Genetic and pharmacological inhibition of METTL3 protects renal function and ameliorates AKI through multiple mechanisms, including the regulation of inflammation, pyroptosis, and ferroptosis, suggesting that targeting the METTL3-mediated axis may provide novel intervention strategies for AKI treatment. Several METTL3 inhibitors have demonstrated therapeutic potential in the treatment of AKI. For example, STM2457, a METTL3 inhibitor, protects against lead acetate-induced renal injury and inflammation (63). Furthermore, STM2457 ameliorates tubular injury and ferroptosis in folic acid -induced AKI mice by inhibiting METTL3 (79). Cpd-564 is a METTL3 inhibitor that offers superior protection against cisplatin- and I/R-induced renal injury and inflammation compared to the previously identified METTL3 inhibitor S-adenosyl-L-homocysteine (50). Berberine is a natural alkaloid with multiple pharmacological effects, including anti-inflammatory, antioxidant, and immunomodulatory properties. Berberine ameliorates pyroptosis and AKI by inhibiting METTL3 expression (78). Another important “eraser”, METTL14, has been shown to be upregulated in AKI, promoting the progression of the condition (52, 53). However, other studies have shown that METTL14 can inhibit ferroptosis, improve renal function, and ameliorate renal injury in mice with AKI (54, 55). Its precise role in AKI remains unclear, and further research is required to elucidate its function in AKI to ensure the safety and efficacy of METTL14-targeted therapies for AKI.

FTO, a key “eraser” of m6A modifications, is frequently downregulated in AKI. Overexpression of FTO can ameliorate AKI through mechanisms such as suppression of inflammation, inhibition of apoptosis, and regulation of autophagy; consequently, FTO activators represent a viable therapeutic strategy for AKI. Studies have reported that curcumin-loaded bone marrow-derived stem cell (BMSC) exosomes can upregulate FTO, inhibit cell apoptosis, inflammation, and oxidative stress, and ameliorate CLP-induced AKI (76). Another m6A modification “eraser”, ALKBH5, has been found to be downregulated in AKI; studies have reported that Alkbh5 knockout mice exhibited reduced I/R-induced AKI (58). Further studies have reported that knockdown of Alkbh5 significantly reduced cisplatin-induced ferroptosis, thereby ameliorating renal injury (59). Additionally, the ALKBH5 inhibitor IOX1 has been reported to increase the recruitment of renal Tregs, reduce the infiltration of neutrophils and macrophages, and exert a protective effect against I/R-induced AKI (58). These studies suggest that ALKBH5 inhibition represents a viable therapeutic strategy for AKI. Although both FTO and ALKBH5 are m6A modification “erasers”, they exert apparently opposed effects in AKI; the underlying mechanisms remain unclear and may be related to their respective target genes.

There is a wide variety of m6A modification “readers”, and different “readers” exert distinct effects on gene expression; consequently, their roles in AKI are complex. m6A modification relies on “readers” to exert its functions. Therefore, enhancing or inhibiting the expression of “readers” according to their respective functions may represent a viable therapeutic strategy for AKI. Furthermore, combining METTL3 inhibitors with “reader” inhibitors for the treatment of AKI may enhance both safety and efficacy.

Although targeting m6A modification represents a promising therapeutic strategy for AKI, achieving kidney-specific regulation of this epitranscriptomic mechanism without inducing systemic or off-target effects remains a significant challenge for clinical translation. The use of highly targeted delivery systems offers a powerful approach to address this hurdle. Exosomes are extracellular vesicles (EVs) released by various cell types and can serve as natural carriers for proteins, lipids, and nucleic acids. Emerging evidence indicates that exosomes can act as delivery vehicles to modulate m6A modification in diverse diseases. For instance, exosomes have been used to deliver METTL14 to osteoclasts, increasing the m6A methylation level of NFATc1 to correct osteoclast-induced bone resorption (110). Exosomes can also deliver WTAP siRNA to ischemic myocardial tissue, achieving specific gene knockdown and conferring myocardial protection (111). Similarly, exosomal delivery of si-FTO synergistically alleviates dopaminergic neuron death in Parkinson’s disease through m6A-dependent regulation of ATM mRNA (112). In the context of kidney disease, extracellular vesicles have been reported to deliver therapeutic macromolecules to injured renal cells to alleviate AKI (113). Specifically, Yang et al. demonstrated that adipose-derived stem cell-derived extracellular vesicles (ADSC-EVs) carry circular RNA circITCH into kidney tissues and cells, promoting CRBN expression in an m6A-dependent manner to protect TECs. Nanoparticle-mediated targeted drug delivery also represents a promising strategy. Numerous studies have reported the application of nanoparticle-based delivery systems in AKI treatment (114–116). Therefore, leveraging nanoparticles to achieve targeted regulation of m6A modification in the kidney may represent a viable and innovative therapeutic approach.

The characteristic changes in m6A modification and its regulatory factors during the onset and progression of AKI suggest their potential utility as novel biomarkers. Several studies have reported significantly altered levels of key regulatory factors—including METTL3, METTL14, ALKBH5, and FTO—in AKI, indicating their diagnostic potential. Moreover, post-AKI renal repair and fibrosis are closely linked to dynamic changes in m6A modification. For instance, METTL3-mediated m6A hypermethylation disrupts mitochondrial homeostasis by inhibiting YME1L1 transcription, thereby promoting the transition from AKI to CKD. This finding suggests that persistently elevated METTL3 expression may serve as an indicator of poor renal repair outcomes (75). Despite these promising associations, detecting the expression of m6A regulatory factors directly within the kidney remains clinically challenging. A key future direction, therefore, is to investigate whether the expression levels of these m6A regulators in circulating blood can serve as non-invasive biomarkers for the diagnosis and prognosis of AKI.

## 7 Conclusion and outlook

Current evidence clearly indicates that m6A modification is not a static background marker but rather a central regulatory hub for dynamic responses. Under AKI stress conditions, m6A precisely regulates the expression of numerous effector molecules in key pathways such as apoptosis, autophagy, and inflammatory responses, and is deeply involved in the biological processes that determine the fate of TECs (e.g., survival, repair, or programmed cell death), thereby significantly influencing the severity of AKI and renal repair outcomes. This provides a novel perspective on the molecular nature of AKI that extends beyond traditional gene transcription. Although breakthrough progress has been made in this field, the field remains largely in its infancy, with many key questions requiring further exploration. Currently, research on m6A modification and AKI remains confined to animal and cellular models, and substantial challenges remain in advancing its translation into clinical practice.

First, current studies have inherent limitations and require further clarification; for example, the “erasers” FTO and ALKBH5 exert opposing effects in AKI, and the underlying mechanisms remain unclear—it is uncertain whether this is because ALKBH5 does not act as an “eraser” in AKI but rather functions in an m6A modification-independent manner. Furthermore, METTL14 appears to exert both pro- and anti-AKI effects simultaneously, necessitating further research to elucidate its context-dependent regulatory networks. Second, targeting m6A regulatory elements—for example, through the development of specific small-molecule inhibitors or activators—offers a novel approach to AKI intervention. However, achieving kidney- or cell-type-specific targeted delivery to avoid the side effects associated with systemic interventions remains a major challenge for future drug development.

In summary, research into m6A modification presents a paradigm-shifting opportunity for understanding the mechanisms of AKI and developing prevention and treatment strategies. With continuous advances in research tools and increasingly deepening interdisciplinary collaboration, elucidating the precise regulatory networks of m6A in kidney disease will undoubtedly translate precision diagnostics and targeted therapies based on epigenetic regulation from concept to reality, ultimately improving the clinical prognosis of patients with AKI.
